# Supplementary material for: Identification of photoperiod-induced specific miRNAs in the adrenal glands of Sunite sheep (Ovis aries)
Source: Front Vet Sci. 2022 Jul 22;9:888207. doi: 10.3389/fvets.2022.888207 (PMC9354845; doi:10.3389/fvets.2022.888207)
Supplement: Supplementary file 1 [file Data_Sheet_1.ZIP › Supplementary file/miRDeep2 file.zip!/miRDeep2 file/Know_Structure/SPLP42A1/oar-mir-655.pdf]

miRBase precursor : oar-mir-655
Total read count : 2823
oar-miR-655-5p read count : 6
oar-miR-655-3p read count : 2817
remaining reads : 0

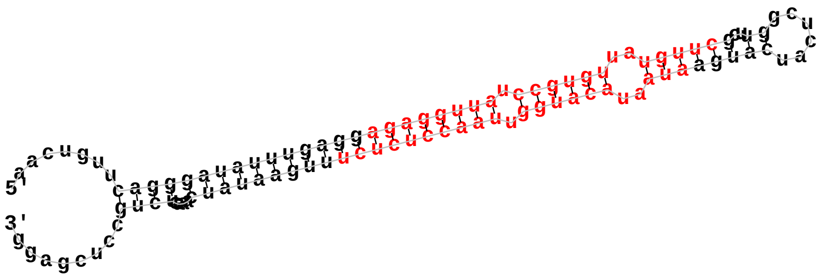

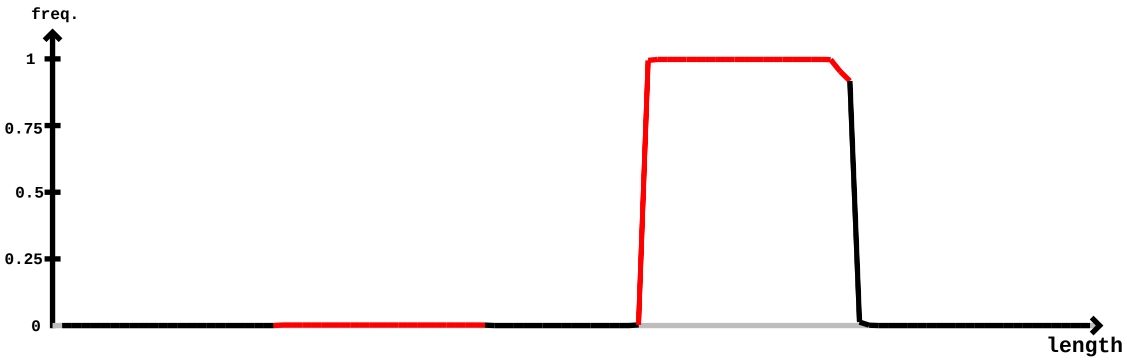


```
                     oar-miR-655-5p
                                                        oar-miR-655-3p
5'- aacuguucagggauauuugaggagagguuauccguguuauguucgcuuggcucaucaugaauaauacaugguuaaccucucuuugaauaucaaacucugccucgagg   -3'   exp
    ...........(((((((((((((((((((.((((((..((((((..((......))))))))..)))))).)))))))))))))))))))...(((......))).   reads   mm   sample
    ......................agagguuauccguguuaugu.................................................................   1       0    seq
    ......................agagguuauccguguuauguuc...............................................................   5       0    seq
    ...........................................................aauaauacaugguuaaccucu...........................   6       0    seq
    ...........................................................aauaauacaugguuaaccucucu.........................   1       0    seq
    ............................................................auaauacaugguuaaccuc............................   2       0    seq
    ............................................................auaauacaugguuaaccucu...........................   120     0    seq
    ............................................................auaauacaugguuaaccucuc..........................   100     0    seq
    ............................................................auaauacaugguuaaccucucu.........................   2541    0    seq
    ............................................................auaauacaugguuaaccucucuu........................   33      0    seq
    ............................................................auaauacaugguuaaccucucuuu.......................   4       0    seq
    .............................................................uaauacaugguuaaccucucu.........................   10      0    seq
```
